# Temporal Muscle and Stroke—A Narrative Review on Current Meaning and Clinical Applications of Temporal Muscle Thickness, Area, and Volume

**DOI:** 10.3390/nu14030687

**Published:** 2022-02-06

**Authors:** Masahito Katsuki, Yukinari Kakizawa, Akihiro Nishikawa, Yasunaga Yamamoto, Toshiya Uchiyama, Masahiro Agata, Naomichi Wada, Shin Kawamura, Akihito Koh

**Affiliations:** 1 Department of Neurosurgery, Suwa Red Cross Hospital, Suwa 392-8510, Nagano, Japan; ktk1122nigt@gmail.com (M.K.); aki.west@gmail.com (A.N.); yamamotoyasunaga@gmail.com (Y.Y.); u_tosh@yahoo.co.jp (T.U.); massa_chiba_3@yahioo.co.jp (M.A.); yosouuemon@gmail.com (N.W.); 2 Department of Neurosurgery, Itoigawa General Hospital, Itoigawa 941-0006, Niigata, Japan; massa_chiba_1@yahoo.co.jp (S.K.); massa_chiba_2@yahoo.co.jp (A.K.)

**Keywords:** frailty, muscle volume, nutritional status, prognostic factor, sarcopenia, skeletal muscle mass, stroke, temporal muscle thickness

## Abstract

Background: Evaluating muscle mass and function among stroke patients is important. However, evaluating muscle volume and function is not easy due to the disturbances of consciousness and paresis. Temporal muscle thickness (TMT) has been introduced as a novel surrogate marker for muscle mass, function, and nutritional status. We herein performed a narrative literature review on temporal muscle and stroke to understand the current meaning of TMT in clinical stroke practice. Methods: The search was performed in PubMed, last updated in October 2021. Reports on temporal muscle morphomics and stroke-related diseases or clinical entities were collected. Results: Four studies reported on TMT and subarachnoid hemorrhage, two studies on intracerebral hemorrhage, two studies on ischemic stroke, two studies on standard TMT values, and two studies on nutritional status. TMT was reported as a prognostic factor for several diseases, a surrogate marker for skeletal muscle mass, and an indicator of nutritional status. Computed tomography, magnetic resonance imaging, and ultrasonography were used to measure TMT. Conclusions: TMT is gradually being used as a prognostic factor for stroke or a surrogate marker for skeletal muscle mass and nutritional status. The establishment of standard methods to measure TMT and large prospective studies to further investigate the relationship between TMT and diseases are needed.

## 1. Introduction

Stroke is a widely known cause of disability [1]. Stroke also increases the risk of skeletal muscle loss [2]—sarcopenia—which contributes to further disability related to stroke [3]. Furthermore, pre-stroke sarcopenia is also associated with poor functional outcomes [4,5]. Therefore, evaluating muscle mass and function among stroke patients is important [6,7], and aggressive nutrition therapy [8,9,10], deprescribing [11], and rehabilitation [12,13] re applicable for those with stroke, as well as those at high risk for muscle loss.

Measuring skeletal muscle mass and function is an evolving parameter for the clinical evaluation of physiological conditions [14]. The gold standard to evaluate sarcopenia are muscle function tests such as the gait speed test and the grip strength test, according to the European Working Group on Sarcopenia in Older People (EWGSOP), EWGSOP2, and the Asian Working Group for Sarcopenia (AGWS) [15,16]. However, measuring muscle function such as grip strength and gait speed sometimes cannot be performed because stroke patients often have disturbances of consciousness, are sedated, are resting due to surgical treatment, or experience paresis. Therefore, an alternative method to evaluate muscle mass and function is needed.

Recently, temporal muscle thickness (TMT) on computed tomography (CT) images or magnetic resonance images (MRI) has been introduced as a novel surrogate marker with which to measure muscle mass [17], function [18], and nutritional status [19,20]. CT and MRI are routinely performed for stroke patients, and TMT measurement is easier than other methods such as quantitative measurements of the cross-sectional skeletal muscle area at the third lumbar vertebra using CT imaging, which is known to be significantly correlated with whole-body muscle [16]. Therefore, TMT is attractive as an alternative method to evaluate muscle mass and function for stroke patients. The first purpose of the narrative literature review that we present herein is to investigate reports on TMT and stroke. The second purpose is to understand the current meaning of TMT in clinical stroke practice. In addition to TMT [21,22], we also examined temporal muscle area (TMA) [21,23,24] and temporal muscle volume (TMV) [9] as novel TM-related surrogate markers for skeletal muscle mass.

## 2. Materials and Methods

Studies regarding TMT and stroke were examined. The search was performed in PubMed, last updated in October 2021, using the terms “stroke” OR “intracerebral hemorrhage (ICH)” OR “subarachnoid hemorrhage (SAH)” OR “cerebral infarction” OR “rehabilitation” OR “sarcopenia” OR “frailty” OR “nutrition” AND “temporal muscle thickness”. The PubMed search resulted in a total of 73 articles. We systematically read through the abstracts of all original articles available in English. We included studies on the association between stroke and TMT, with a sample size of around 50 cases and appropriate statistical analyses. We also checked through the lists of references to complete our collection of studies. All the authors verified the correct transcription of the data to our manuscript. Finally, we included eight studies related to stroke in our review.

## 3. Results

**Figure 1:**
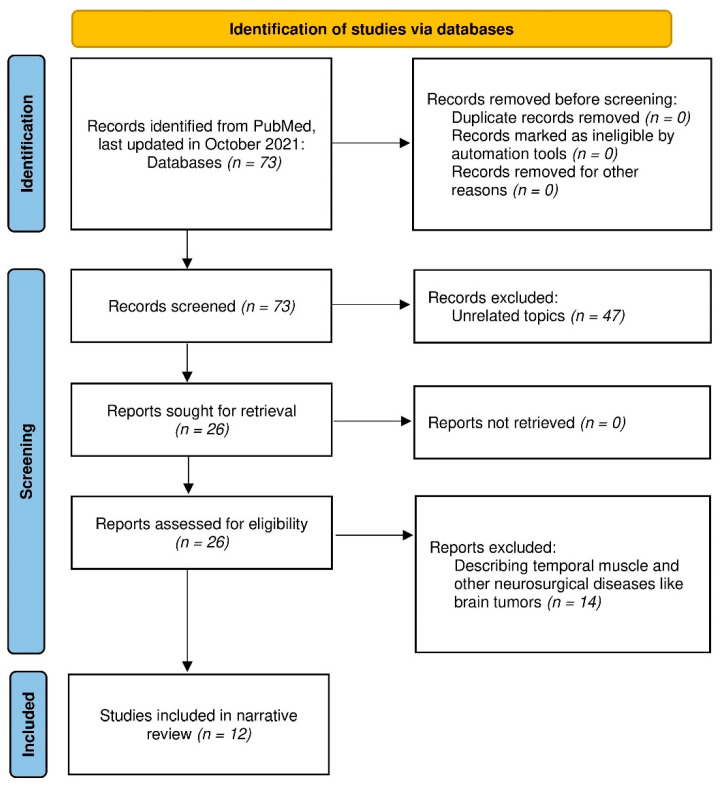
PRISMA 2020 flow diagram for this review.

Four studies reported the association between TMT and SAH [9,21,23,24], two studies between TMT and ICH [25,26], and two studies between TMT and ischemic stroke [27,28]. The other four studies described the standard TMT values [18,29] and the relationship between TMT and nutritional status [19,20]. The Preferred Reporting Items for Systematic Reviews and Meta-Analyses (PRISMA) 2020 flow diagram [30] for systematic review is shown as Figure 1.

### 3.1. Temporal Muscle and SAH

Katsuki et al. first reported TMT as a prognostic factor for SAH outcomes in 2019, investigating 49 SAH patients over 75 years of age who were treated by clipping with craniotomy [21]. TMT was measured on CT images on admission, using Aquilion ONE (Canon Medical Systems Corporation, Tochigi, Japan) with 0.5 × 0.5 x 1.0 mm voxels. The slice thickness was reconstructed to 5 mm. The window width was adjusted to 300 Hounsfield units and the window level was adjusted to 20 Hounsfield units. TMT was measured bilaterally perpendicular to the long axis of the temporal muscle at a slice 5 mm above the orbital roof using SYNAPSE V 4.1.5 imaging software (Fujifilm Medical, Tokyo, Japan). Then, the averages of the left and right of the TMTs were used. The method to measure TMT on CT was thereby defined. Katsuki et al. then performed univariate analysis regarding TMT and functional outcome at six months. The study was preliminary, but the study suggested that greater TMT was related to favorable outcomes among elderly SAH.

Katsuki et al. next investigated the relationship between temporal muscle and Hunt and Kosnik grade on admission and functional outcome at six months [23]. They examined 298 all age-group patients, and all patients were treated by endovascular coiling. They revealed that the Hunt and Kosnik grade on admission and functional outcome were related to TMT and TMA. TMA was measured manually by tracing the outline of the temporal muscle on the same CT slice as that used for measuring TMT. Notably, this study suggests that TMT and TMA are related to both the severity of SAH and functional outcome regardless of age, not only for the elderly.

They then investigated 127 SAH patients under 75 years of age who were treated by clipping [24]. They examined the cut-off values for the functional outcomes. Receiver operating characteristic analysis found that the threshold of TMT was 4.9 mm in women and 6.7 mm in men, and that of TMA was 193 mm^2^ in women and 333 mm^2^ in men, which were the cut-off values for the functional outcomes among SAH patients under 75 years of age.

Onodera et al. [9] examined TMV using volume rendering software (Ziostation 2 version 2.9.5.1, Ziosoft, Tokyo), because TMT may be less reproducible. They investigated 60 SAH patients and measured TMV on the CT images at admission and two weeks after aneurysm treatment. Patients whose TMV had decreased by ≥20% were classified into the “atrophy group,” whereas those whose TMV had decreased by <20% were classified into the “maintenance group.” Their study showed that the food intake score and the functional outcome were significantly more positive in the TMV maintenance group than the TMV atrophy group. Therefore, this study suggests the importance of early high-protein administration to maintain TMV in the acute term (Table 1).

### 3.2. Temporal Muscle and ICH

Katsuki et al. examined 75 ICH patients treated by endoscopic hematoma removal and investigated the factors related to the functional outcome [25]. They revealed that lower total protein level was related to poor outcomes at six months. In addition, they mentioned TMA as an indicator of nutrition, but TMA itself was not significantly related to the outcome (*p* = 0.08). However, they suggested that low nutritional status, indicated by lower total protein level and low TMA altogether, seemed to be associated with poor outcomes.

Gomes et al. examined 24 post-hemorrhagic stroke patients in the chronic stage and tested bite force and TMT [26]. Maximum molar bite force was verified using a digital dynamometer. TMT was measured using ultrasound images obtained at rest and during maximal voluntary contraction of the masseter and temporalis muscles. The TMT on the unaffected side was larger than on the affected side. This study first focused on the functional and morphological changes in the stomatognathic system after a hemorrhagic stroke. The clinical meaning of these changes was investigated (Table 2).

### 3.3. Temporal Muscle and Stroke

Sakai et al. [27] investigated 70 acute cerebral infarction patients’ TMT on the T2-weight MR image and functional oral intake scales. They revealed that TMT was a significant explanator of dysphagia severity following acute ischemic stroke, along with age and the National Institute of Health Stroke Scale score. The measuring method of TMT using T2-weighted images was similar to the previous report from Furtner et al. using T1-weighted images [31]. They first reported the association between TMT and ischemic stroke-related dysphagia in the acute term.

Nozoe et al. [28] examined 289 acute elderly stroke patients and investigated TMT on CT images as an indicator of sarcopenia risk and its relationship with the functional outcome at three months. They found that sarcopenia risk was independently associated with TMT in older patients with acute stroke. However, TMT was not independently related to the functional outcome (Table 3).

### 3.4. Standard Values of TMT

Steindl et al. [18] investigated a 624-individual MRI dataset to establish standard reference values of TMT on T1-weighted images. The cohort consisted of two MRI repositories: The Enhanced Nathan Kline Institute-Rockland Sample [32]; and the Designed Database of MR Brain Images of Healthy Volunteers [33]. TMT was measured on isovoxel (1 × 1 × 1 mm^3^) T1-weighted MR images perpendicular to the long axis of the temporal muscle on an axial plane, which was oriented parallel to the anterior commissure-posterior commissure line. They also examined 422 healthy volunteers and 130 cases as a prospective validation cohort and found that TMT and grip strength were correlated. This was the first report to validate the relationship between the TMT and grip strength, namely muscle function, prospectively.

Katsuki et al. [29] investigated a database of 360 Japanese individuals’ brain check-ups obtained by MRI. They measured TMT in the same way previously reported in [18] to obtain standard values of TMT among Japanese individuals. They compared their result to Steindl’s results to obtain the racial difference, but the background of the participants differed. They did not perform any muscle function test, so further investigation is needed (Table 4).

### 3.5. TMT and Nutritional Status

Hasegawa et al. [20] investigated 73 elderly individuals to measure their TMT using ultrasonography and nutritional status assessed with anthropometric measurements and laboratory tests. Arm circumference (AC) was measured in the middle of the non-dominant upper arm using a measuring tape. Arm muscle circumference (AMC) was calculated based on the standard procedure using the following formula: AMC (cm) = AC (cm)−π x the triceps skinfold thickness (cm) [34]. Calf circumference (CC) was measured with a tape at the maximum girth of the right calf with the leg in a lying position. TMT was strongly correlated with CC and AMC. However, there were no strong correlations with serum protein levels, nor was fat mass evaluated in the triceps skinfold thickness. They also examined the reliability to measure TMT using ultrasonography; the inter-rater reliability was 0.99.

Hasegawa et al. also performed a prospective study [19]. The study aimed to examine whether a change in TMT evaluated by the ultrasonography was directly correlated with energy adequacy, and to determine the cut-off value of a change in TMT to detect energy inadequacy. They investigated 48 bedridden elderly patients and revealed that percentage change in TMT was significantly correlated with energy adequacy. They suggested that the assessment of TMT changes could be helpful for performing better nutritional therapy (Table 5).

## 4. Discussion

We herein reviewed reports on TMT and stroke. TMT is useful as a prognostic marker for SAH, ICH, and dysphagia after stroke. It also indicates nutritional status and risk of sarcopenia. As the number of reports on TMT and stroke has been increasing rapidly in recent years, we believe that TMT is one of the important factors in clinical practice. In addition to this review, we discussed the TMT measurement method and TMT use in other neurosurgical practices.

### 4.1. TMT Measurement Method

A standard TMT measurement method has not been established. Old reports used volume rendering software [35,36], and Onodera et al. also used a similar approach [9] to measure TMV, but not TMT. Then, Furtner et al. established TMT measurement using T1-weighted MR images. They measured TMT perpendicular to the long axis of the temporal muscle at the level of the orbital roof [14,17,18,37,38]. This method is widely used, but low accessibility to MRI in routine work is a problem. Sakai et al. [27] used T2-weighted MR images, rather than T1-weighted images. The difference between the T1- and T2-weighted images should be discussed. Katsuki et al.first defined TMT and TMA on CT images [21]. CT is more accessible than MRI, so TMT measurement on CT seems better for routine clinical work. Hasegawa et al. used ultrasonography (M-Turbo; SonoSite, Bothell, WA, USA) to measure TMT at 4 cm from the eyelid and 2 cm above the reference line, which was the orbitomeatal line [20]. Ultrasonography is not so reproducible, but their study reported that TMT measurement by ultrasonography is reliable.

As described above, there are some ways to measure temporal muscle morphomics, including TMT, TMA, and TMV. Easiness and high reproducibility are needed to establish a standard method. Further study on the measurement method is desirable. 

### 4.2. Temporal Muscle in Other Neurosurgical Practice

The first report on the temporal muscle as a prognostic factor in neurosurgical practice evaluated the operative risk in non-syndromic craniosynostosis in 2013 [36]. The authors used volume rendering software to assess the temporal fat pad. Since this report, there have been several papers on the temporalis muscle and prognosis, especially in brain tumors. There are several reports on overall survival and temporal muscle in glioblastoma [38,39,40,41,42,43,44,45], metastatic brain tumor [31,46,47], and primary central nervous system lymphoma [37,48]. As in reports on TMT and stroke, all of these reports used temporal muscle to indicate nutritional status and skeletal muscle mass volume. The greater the temporal muscle, the better the outcome, probably due to better nutritional status and more skeletal muscle mass. Furthermore, deep learning-based quantification of TMA has been reported [49], so it is expected that TMA measuring will be widely performed. 

### 4.3. Limitations

As described above, TMT is now attractive, and many studies have been performed, but some issues should be addressed. First, most of the studies were retrospective, so further prospective study is needed. Second, the sample sizes were small, so studies with large sample sizes are desirable. Third, a standard TMT measurement method has not been established, and several methods can be used, such as MRI, CT, and ultrasonography. A standard approach to measuring TMT is needed. Fourth, the direct mechanism of why large temporal muscle relates to favorable prognosis has not been clarified. The true mechanism between TMT and outcomes should be discussed from several perspectives, such as rehabilitation, nutrition, frailty, deglutition, or basic medicine. Some of the problems may be resolved as TMT measurements are routinely taken, thereby tracking time-course changes.

## 5. Conclusions

TMT seems to be useful surrogate marker for skeletal muscle volume and function, and is a potential prognostic factor. Research on the association between stroke and TMT is increasing. Further research is needed to establish the usefulness of TMT.

## Figures and Tables

**Table 1:** Previous reports on the association between temporal muscle and SAH.

Author	Year	Number of Cases	Abstract
Katsuki [21]	2019	49	High TMT was related to favorable outcomes among elderly SAH.
Katsuki [23]	2020	298	TMT and TMA were related to Hunt and Kosnik grade and functional outcome at six months after endovascular coiling, regardless of age.
Katsuki [24]	2021	127	The threshold of TMT was 4.9 mm in women and 6.7 mm in men, and that of TMA was 193 mm^2^ in women and 333 mm^2^ in men, which were the cut-off values for the functional outcomes at six months among SAH patients under 75 years of age.
Onodera [9]	2021	60	The food intake score and the functional outcome at discharge were significantly more positive in the TMV maintenance group than the TMV atrophy group after SAH.

Abbreviations: SAH: subarachnoid hemorrhage; TMA: temporal muscle area; TMT: temporal muscle thickness; TMV: temporal muscle volume.

**Table 2:** Previous reports on the association between temporal muscle and ICH.

Author	Year	Number of Cases	Abstract
Katsuki [25]	2019	75	Low nutritional status, indicated by low total protein level and low TMA altogether, seemed to be associated with the poor functional outcomes at six months after endoscopic hematoma removal.
Gomes [26]	2021	24	TMT on the unaffected side was greater than on the affected side after a hemorrhagic stroke.

Abbreviations: ICH: intracerebral hemorrhage; TMA: temporal muscle area; TMT: temporal muscle thickness.

**Table 3:** Previous reports on the association between temporal muscle and stroke.

Author	Year	Number of Cases	Abstract
Sakai [27]	2021	70	TMT was a significant explanator of dysphagia severity following acute ischemic stroke.
Nozoe [28]	2021	289	Sarcopenia risk was independently associated with TMT in older patients with acute stroke, but TMT was not independently related to the functional outcome.

Abbreviations: TMT: temporal muscle thickness.

**Table 4:** TMT and nutritional status.

Author	Year	Number of Cases	Abstract
Steindl [18]	2020	1175	Standard values of TMT were investigated, and TMT and grip strength were correlated.
Katsuki [29]	2021	360	Standard values of TMT were investigated among Japanese individuals who underwent brain check-ups.

Abbreviations: TMT: temporal muscle thickness.

**Table 5:** TMT and nutritional status.

Author	Year	Number of Cases	Abstract
Hasegawa [20]	2019	73	TMT was strongly correlated with CC and ACM. However, there were no strong correlations with serum protein levels, nor was fat mass evaluated in the triceps skinfold thickness.
Hasegawa [19]	2021	48	TMT changes were directly correlated with energy adequacy in bedridden older adults.

Abbreviations: ACM: arm muscle circumference; CC: Calf circumference; TMT: temporal muscle thickness.

## Data Availability

Not applicable.
